# Mixed deep learning and natural language processing method for fake-food image recognition and standardization to help automated dietary assessment

**DOI:** 10.1017/S1368980018000708

**Published:** 2018-04-06

**Authors:** Simon Mezgec, Tome Eftimov, Tamara Bucher, Barbara Koroušić Seljak

**Affiliations:** 1 Jožef Stefan International Postgraduate School, Ljubljana, Slovenia; 2 Computer Systems Department, Jožef Stefan Institute, Jamova cesta 39, Ljubljana 1000, Slovenia; 3 Institute of Food, Nutrition and Health (IFNH), ETH Zürich, Zürich, Switzerland; 4 School of Health Sciences, Faculty of Health and Medicine, Priority Research Centre in Physical Activity and Nutrition, The University of Newcastle, Callaghan, Australia

**Keywords:** Fake food buffet, Food replica, Food image recognition, Food matching, Food standardization

## Abstract

**Objective:**

The present study tested the combination of an established and a validated food-choice research method (the ‘fake food buffet’) with a new food-matching technology to automate the data collection and analysis.

**Design:**

The methodology combines fake-food image recognition using deep learning and food matching and standardization based on natural language processing. The former is specific because it uses a single deep learning network to perform both the segmentation and the classification at the pixel level of the image. To assess its performance, measures based on the standard pixel accuracy and Intersection over Union were applied. Food matching firstly describes each of the recognized food items in the image and then matches the food items with their compositional data, considering both their food names and their descriptors.

**Results:**

The final accuracy of the deep learning model trained on fake-food images acquired by 124 study participants and providing fifty-five food classes was 92·18 %, while the food matching was performed with a classification accuracy of 93 %.

**Conclusions:**

The present findings are a step towards automating dietary assessment and food-choice research. The methodology outperforms other approaches in pixel accuracy, and since it is the first automatic solution for recognizing the images of fake foods, the results could be used as a baseline for possible future studies. As the approach enables a semi-automatic description of recognized food items (e.g. with respect to FoodEx2), these can be linked to any food composition database that applies the same classification and description system.

Measuring dietary behaviour using traditional, non-automated, self-reporting technologies is associated with considerable costs, which means researchers have been particularly interested in developing new, automated approaches. There is a clear need in dietary assessment and health-care systems for easy-to-use devices and software solutions that can identify foods, quantify intake, record health behaviour and compliance, and measure eating contexts. The aim of the present study was to test the combination of an established and validated food-choice research method, the ‘fake food buffet’ (FFB), with a new food-matching technology to automate the data collection and analysis.

The FFB was developed as an experimental method to study complex food choice, meal composition and portion-size choice under controlled laboratory conditions. The FFB is a selection of very authentic replica-food items, from which consumers are invited to choose. The FFB method was validated by a comparison of meals served from real and fake foods^(^ ^1^ ^)^. The food portions served from the fake foods correlated closely with the portions served from the real foods^(^ ^1^ ^)^. Furthermore, significant correlations between the participants’ energy needs and the amounts served were found in several studies^(^ ^1^ ^–^ ^4^ ^)^. It has also been shown that people who selected foods for an entire day from an FFB were able to closely match their dietary requirements^(^ ^5^ ^)^.

In a typical FFB study, the experimenters choose fake foods and set up a buffet. The participants receive instructions, which can contain the experimental intervention, and are then invited to select foods, choose portions of foods to assemble meals^(^ ^2^ ^,^ ^3^ ^)^ or even set a diet for a day^(^ ^5^ ^)^. The experimenter then analyses the choice. Similar protocols and the same fake foods were used for experiments in different countries (i.e. Germany, Switzerland, the UK and Australia). Currently, the FFB study procedure still has several ‘analogue’ components. After the participants select the meals, a photograph is taken, the foods are separated manually, each food is weighed, and the researcher calculates the nutritional values for the selected fake foods. This process would benefit from automation. All the consumer choices are recorded and additional fake-food images are available for the aims of the research.

The first step of the automation process is to recognize the fake-food and fake-drink items present in these images. Due to the nature not only of the fake-food and fake-drink items, but also of food and drink items in general, this is a particularly challenging computer vision problem. Differentiating between different food or drink items (henceforth ‘food items’) can sometimes be challenging even for the human eye. The issue is that different food items can appear to be very similar and the same food item can appear to be substantially different on different images because of a variety of factors, such as image quality, illumination, the amount of noise present in the image, the way in which the food item was prepared and served, etc.

The next step is to match the fake-food items recognized in the image to food composition data, which are detailed sets of information on the nutritionally important components of foods, providing values for the energy and nutrients, including protein, carbohydrates, fat, vitamins and minerals, and for other important food components, such as fibre, etc. The data are presented in food composition databases (FCDB). The process of semi-automatic food matching is a crucial part of an automated dietary assessment.

In the current paper, we present results of a study performed with the objective to develop an automated dietary assessment that consists of two main activities: (i) automatically recognizing fake-food and fake-drink items from photos; and (ii) automatically assigning (matching) recognized items to their compositional data. Using this approach, the dietary assessment can be performed much more quickly and, in many cases, also more accurately than if performed manually.

The paper proceeds as follows. In the next section we present relevant work on the FFB, food image recognition and food matching. Thereafter we introduce the methodology applied in the present study to an automated dietary assessment. Next we show how this methodology was applied to fake foods and present the results of the evaluation. Finally, we discuss the results and present some ideas for future work.

## Relevant work

### The fake food buffet

Replica-food models such as the Nasco food models^(^ ^6^ ^)^ have traditionally been used in dietary assessment as portion-size estimation aids and for educational purposes. However, only recently have food-replica models been validated and used for experimental studies in food-choice and consumer behaviour research^(^ ^1^ ^)^. The FFB method has, for example, been used to investigate environmental influences such as plate size^(^ ^3^ ^)^, vegetable variety^(^ ^7^ ^,^ ^8^ ^)^ in food choice, or the effect of the nutritional information and labels on food choice for a single meal^(^ ^2^ ^,^ ^9^ ^)^ or for an entire day^(^ ^5^ ^)^. Fake foods were also used to investigate health perceptions^(^ ^4^ ^,^ ^10^ ^)^ and social influences and attitudes to food choices^(^ ^11^ ^,^ ^12^ ^)^.

Meanwhile, the FFB is an established research tool within several research facilities worldwide; research institutions in Germany, Switzerland, the UK and Australia are using a similar set of replica foods to address a variety of research questions. However, to date the procedure of carrying out an FFB experiment still involves several manual steps, including identifying and quantifying the foods selected by the study participants, and different research laboratories use different FCDB to calculate the theoretical nutrient contents of the fake foods. The differences in the nutrient profile of the same food between different nutrient databases in different countries might reflect actual differences in the composition of these foods in the different countries. Linking the fake foods to standardized nutrient contents (e.g. an EU database) might remove certain country-specific information (e.g. related to food processing). However, the standardization of the nutrient content calculation would still greatly facilitate international collaboration and the comparison of food portions.

### Food image recognition

Until recently, the approach favoured by most researchers in the field of food image recognition was based on manually defined feature descriptors^(^ ^13^ ^–^ ^15^ ^)^. However, because of the complexity of the features in food images, this approach did not perform well.

Recently, deep learning, a fully automatic machine learning approach, achieved state-of-the-art results in a wide variety of computer vision problems and proved to be most effective for the task of image recognition. It has also been validated in the field of food image recognition multiple times^(^ ^16^ ^–^ ^23^ ^)^. However, to the best of our knowledge, there are no previous solutions that would automatically recognize drinks from images, and the number of food classes in the data sets that have been used so far is very limited – often up to 100 different food types or less. This is why we have introduced an approach that addresses both of these issues^(^ ^24^ ^)^. It is a unique approach due to how the food and drink image data set is built as well as the custom deep learning network used. Using this approach, we have achieved an accuracy of 86·72 % on a new data set containing 520 different food and drink items. However, our approach, as well as most solutions listed above, have a shortcoming: they are incapable of recognizing more than one food item per image. We address this issue in the current paper as we are performing pixel-level classification, which is not limited to any specific number of recognized food items.

The research works described above classify food items into food classes, which can then be linked to FCDB to add compositional information. However, there is another approach to this problem: perform food ingredient recognition and try to directly recognize the food ingredients from the image. This has been presented in a few recent solutions by Chen *et al.* ^(^ ^25^ ^,^ ^26^ ^)^ and Salvador *et al.* ^(^ ^27^ ^)^, which detail the process of recognizing ingredients from food images and then linking them with recipes containing those ingredients.

### Food matching

Matching food items with compositional data can be performed in two ways, by considering either the food descriptors or the food names. Databases on food composition, consumption, allergens, etc. describe food items with descriptors (terms and facets) defined by a classification and indexing system. Several such systems exist (e.g. FoodEx2^(^ ^28^ ^)^, LanguaL^(^ ^29^ ^)^); however, many databases are lacking food descriptors because defining them is a time-consuming task. Therefore, matching food items from different data sources by considering food names is a relevant challenge. The problem of matching food with compositional data through food names is that the same food can have different food names within different data sources (i.e. different FCDB)^(^ ^30^ ^)^. This is because people who express themselves in different ways or have unique writing styles defined the food names. For example, the food item name that results from the food image recognition method depends on the person who developed the method, while the food item name presented in the FCDB depends on the person or company who performed the nutrient analysis and then provided and stored the result. To address this problem, in 2016 we developed a promising method for matching food items to their compositional data using food names and text-similarity measures applied at a word level, which was aimed at matching food items to their compositional data^(^ ^31^ ^)^. Meanwhile, we have extended this method to classify and describe food items considering both food names and food descriptors that are semi-automatically assigned to the food items^(^ ^32^ ^)^.

## Methods

### The fake food buffet

In the current study we used the image data from an FFB experiment in which 124 participants were invited to serve themselves lunch from a buffet with replica foods. Details about the procedures of the experimental study are described elsewhere^(^ ^2^ ^)^. In total, 121 photographs were used (two images were missing, one image was incomplete) and out of the fifty-seven food classes, fifty-five were matched (‘margarine’ was not present in any images and ‘fish sticks’ were present in only one image, which is not enough to train a deep learning model).

### Fake-food image recognition

Food image recognition requires several steps to be performed: image pre-processing, deep learning model training, testing and validation. We are also performing data augmentation in the pre-processing step, by which we are referring to the process of expanding the original image data set by generating additional variants of original images, which is beneficial for deep learning methods as they require as large a data set as possible for increased real-world accuracy^(^ ^33^ ^)^.

#### Image pre-processing

To train a deep learning model on the fake-food images we first needed to manually pre-process the images. The main aim of the pre-processing step is to generate ‘ground-truth’ labels for the food items present in each image, which are later needed for the supervised learning of the deep learning model. Ground truth refers to information that we know is correct; in the case of food images, this means that the labels for each of the food items are reliable. Usually, the simplest approach to generating such labels is labelling each image with one food class (food name) and training a deep learning model in such a way that it returns one text label per image. However, since all the images from the FFB not only contain multiple food items, but have over eleven foods on average, such an approach would be very inaccurate and is therefore not appropriate for this application.

That is why for generating ground-truth data we needed to label not just each image, but each food item present in each image.

As foods often overlap on plates and drinks can obstruct the view of other items, we labelled each food item on a pixel level, which means that the result of this step was a new label image with the same width and height as the input image, only with a single channel as opposed to three channels used in RGB images. This label image contains a class prediction for each individual pixel, so a ‘tomato’ item has all its pixels labelled as ‘tomato’ and its surrounding pixels are labelled as another class.

Since generating such ground-truth labels without significant errors is non-trivial and is one of the main obstacles when trying to design a pixel-level classification solution, we manually segmented each food and drink item in each of the 121 fake-food images. This has resulted in 121 label images with a total of 1393 different food and drink items, each belonging to one of the fifty-five food and drink classes.

After the labelling part, the fake-food data set was randomly split into training (70 % of images), validation (10 %) and testing (20 %) subsets to use for the deep learning model training such that any image was used in only one of the subsets. The food objects are the same across all three subsets, although the selection of food objects differs from image to image. Finally, four different data-augmentation steps were performed on the images in the training subset, as well as their corresponding label images. These steps included: rotating each image by 90°, 180° and 270°; flipping the image horizontally; adding random colour noise; and zooming in on the image so that 25 % of the image’s borders were removed^(^ ^24^ ^)^. It is important to note that while the other data-augmentation steps were performed in the same way on both the fake-food images and the label images, random noise was introduced only to the food images, as the ground-truth labels should not change, even in the presence of noise. The result of the data-augmentation process is therefore seven variations per fake-food image in the training subset. In total, the final fake-food data set with the augmented training subset contains 631 images with 7222 food or drink items (some items were cut off in the zoomed-in image variants). All the fake-food and label images have a resolution of 500 pixels×375 pixels; the reason for the lower resolution is the considerable memory requirements of the deep learning approach used, which is described in the following section.

#### Deep learning model training

We trained the fake food and drink recognition model using deep convolutional neural networks, which are a type of neural network that works in a similar way to human vision: individual neurons react to overlapping regions in the visual field. Specifically, we used fully convolutional networks (FCN) that were introduced in a study by Long *et al.* ^(^ ^34^ ^)^ and represent the state-of-the-art for semantic segmentation. This process segments the input image into separate parts and then classifies each part into an output class; the network does that by performing pixel-level classification. The FCN therefore outputs a pixel map instead of a class text label, and this pixel map contains predictions from the model for each individual pixel of the input image, as opposed to having only one prediction for the entire image. This is important because, as mentioned in the previous section, it is the most accurate way to describe all the food items present in one image. Long *et al.* ^(^ ^34^ ^)^ introduced three FCN variants: FCN-32s, FCN-16s and FCN-8s. The FCN-32s outputs a pixel map based on the predictions from the final layer of the fully convolutional network, which is the standard approach for semantic segmentation networks. The FCN-16s, on the other hand, combines the predictions from the final layer with those from an earlier layer, which contains a more detailed representation of the input image, thus allowing the network to make predictions at a finer grain. Finally, the FCN-8s considers an additional layer when making predictions compared with the FCN-16s, and it is therefore able to segment the input images at the finest grain. This is why, of all the FCN variants available, the FCN-8s is the best performing, making it suitable for food and drink image recognition.

Since it is possible to use deep learning models that are pre-trained on other data sets as a starting point for the model training process, we wanted to use an FCN-8s model that was pre-trained on the PASCAL Visual Object Classes (PASCAL VOC) data set^(^ ^35^ ^)^ to decrease the training time and increase the number of images for training, thus improving the robustness of the final model. However, since this data set contains images from only twenty-one different classes, we needed to modify the FCN-8s network architecture to use it for the recognition of our fifty-six classes (fifty-five fake-food classes and the background class). This was done by adding an extra layer at the end of the deep learning network, which increases the number of output classes from twenty-one to fifty-six. Doing this was necessary to take advantage of the pre-trained network, as otherwise the output layer would have to be retrained from the start.

For the deep learning model training we used the popular deep learning framework Caffe, which was developed by the Berkeley Vision and Learning Center^(^ ^36^ ^)^, and the NVIDIA Deep Learning GPU Training System (NVIDIA DIGITS), which is a graphical user interface built upon Caffe and provides feedback options during the model training process^(^ ^37^ ^)^.

To train the models, we used Adam^(^ ^38^ ^)^ as the solver. Solvers are methods that perform updates to deep neural network parameters in each training epoch with the goal to minimize the loss function, which is the primary quality measure while training the models. The solver is therefore an important part of the deep learning model training process that tunes the model in such a way that it reacts to features in the input images and learns to classify them successfully. Adam is a solver that automatically adapts the learning rate to the parameters. The learning rate defines the rate with which the parameters are changed during the training process; the higher the learning rate, the faster the model converges to the optimal loss value, which speeds up the training. However, the learning rate should not be set too high because the model might then converge to a worse loss value, or not converge at all. It is therefore important to choose an appropriate rate, and we achieved the best results by setting the initial learning rate to 0·0001 and letting Adam automatically adapt this rate during the training.

Since the FCN perform the classification of each individual pixel, their memory requirements are much greater than those of traditional convolutional neural networks where large batches of images can be processed at the same time. Because of this we had to set the software to process only one image at a time, as one image alone completely filled the video random access memory of the graphics processing unit. Additionally, we trained the model for 100 epochs and then selected the final model at the epoch where the loss on the validation subset stopped decreasing, as that signals the moment when the model starts overfitting on the training data. For the model training, we used a single NVIDIA GeForce GTX TITAN X graphics processing unit.

#### Measures

To measure the performance of the trained deep learning model we used the same evaluation measures as Long *et al.* ^(^ ^34^ ^)^, since their study showed that these measures are appropriate to test the FCN models. The measures are based on the standard pixel accuracy and Intersection over Union (IU) measures, including the following.

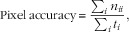




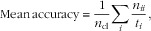




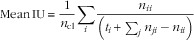

and

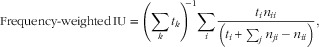

where *n*_cl_ is the number of different classes in the ground-truth labels, *n*_*ij*_ is the number of pixels of class *i* predicted to belong to class *j* and 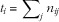 is the total number of pixels of class *i* in the ground-truth labels. We used a Python implementation of these measures^(^ ^39^ ^)^.

### Food matching

To match the food items recognized in the image to an FCDB, we decided to use an approach that involved matching foods by their descriptors and names to achieve the best possible result. However, because most FCDB are lacking food descriptors, we first applied the StandFood method^(^ ^32^ ^)^ to assign FoodEx2 descriptors to the food items in a semi-automated way.

The StandFood method consists of three parts. The first identifies what type of food (raw, derivative, simple or aggregated composite food) is being analysed. This is the classification part that involves a machine learning approach^(^ ^40^ ^)^. The second part describes the food using natural language processing^(^ ^41^ ^)^ combined with probability theory, which results in the list term or FoodEx2 code for the food. For each food item that needs to be described according to FoodEx2, its English name is used. The name is pre-processed by converting it to lowercase letters. Part-of-speech (POS) tagging is used to extract its nouns, adjectives and verbs. The extracted sets are further transformed using lemmatization. Using the extracted nouns, the FoodEx2 data are searched for the names that consist of at least one of the extracted nouns. The resulting list (a subset) is then pre-processed by converting each food item’s name to lowercase letters, applying POS tagging to extract the nouns, adjectives and verbs, and using lemmatization for the extracted sets. Then, the food item that needs to be described according to FoodEx2 is matched with each food item in the resulting list and a weight is assigned to each matching pair. Finally, the pair with the highest weight is the most relevant one, so it is returned together with its food category from FoodEx2. The third part combines the result from the first and the second part by defining post-processing rules to improve the result for the classification part.

The first evaluation of the system was made using 532 foods from the Slovenian FCDB and had an accuracy of 89 % for the classification part and 79 % for the description part. However, 21 % of instances were not correctly described, even though some of these instances were correctly classified. This happens due to the fact that the food items do not exist in FoodEx2, the food items are specific to some cultures, or the POS tagging model that is used for the extraction of the morphological information does not provide nouns, so the search cannot continue.

For the purposes of the current study we extended the StandFood method in the second part. The extension works with cases of food names where nouns cannot be extracted, so instead of using the POS tagging-probability-weighted method^(^ ^42^ ^)^ to find the most relevant match, it switches to the Levenshtein distance^(^ ^43^ ^)^, which can be used as a similarity measure between two textual descriptions.

### The methodology

**Fig. 1:**
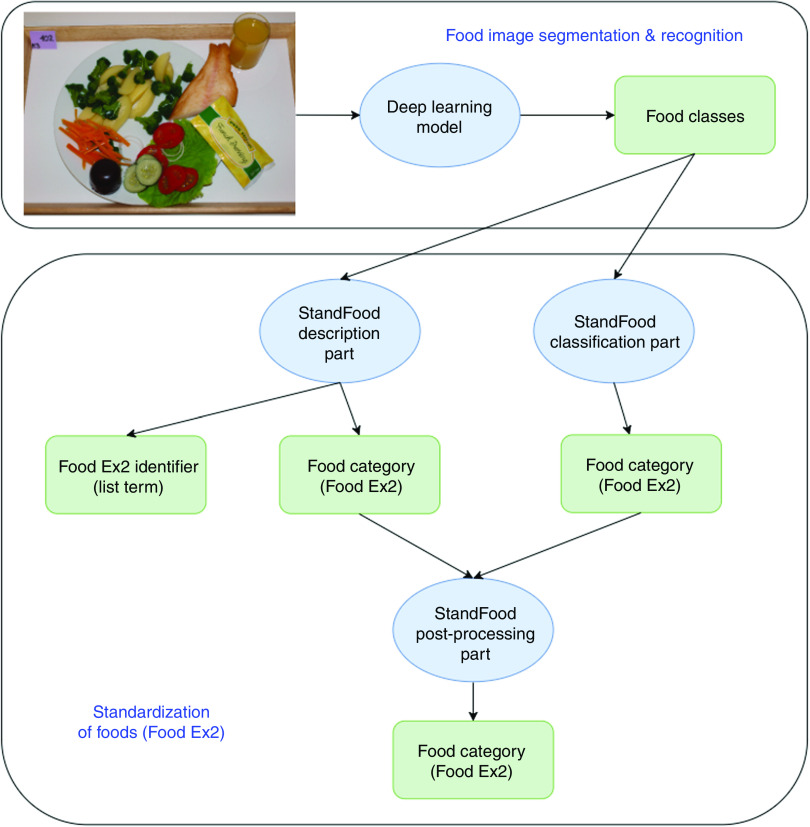
Methodology flowchart. The food image recognition process uses a fake-food image to find classes (names) for all food items in the image. These are then processed by the StandFood method to define the FoodEx2 descriptors of the recognized food items. Once both the food names and descriptors are identified, the recognized fake foods can be matched with compositional data from the food composition database. The final result is a fake-food image standardized with unique descriptors, which enables food intake conversion into nutrient intake and helps the automated dietary assessment

Figure 1 shows a flowchart of the methodology applied in the present study. First, the food image recognition process uses a fake-food image to find the classes (names) of all the food items in the image. These food names are then processed by the StandFood method to define the FoodEx2 descriptors of the recognized food items. Once both the food names and the descriptors are identified, the recognized fake foods can be matched with compositional data from the FCDB. The final result is therefore a fake-food image standardized with unique descriptors, which enables the conversion of food intake into nutrient intake and helps the automated dietary assessment.

## Experimental results

### Results from food image recognition

**Fig. 2:**
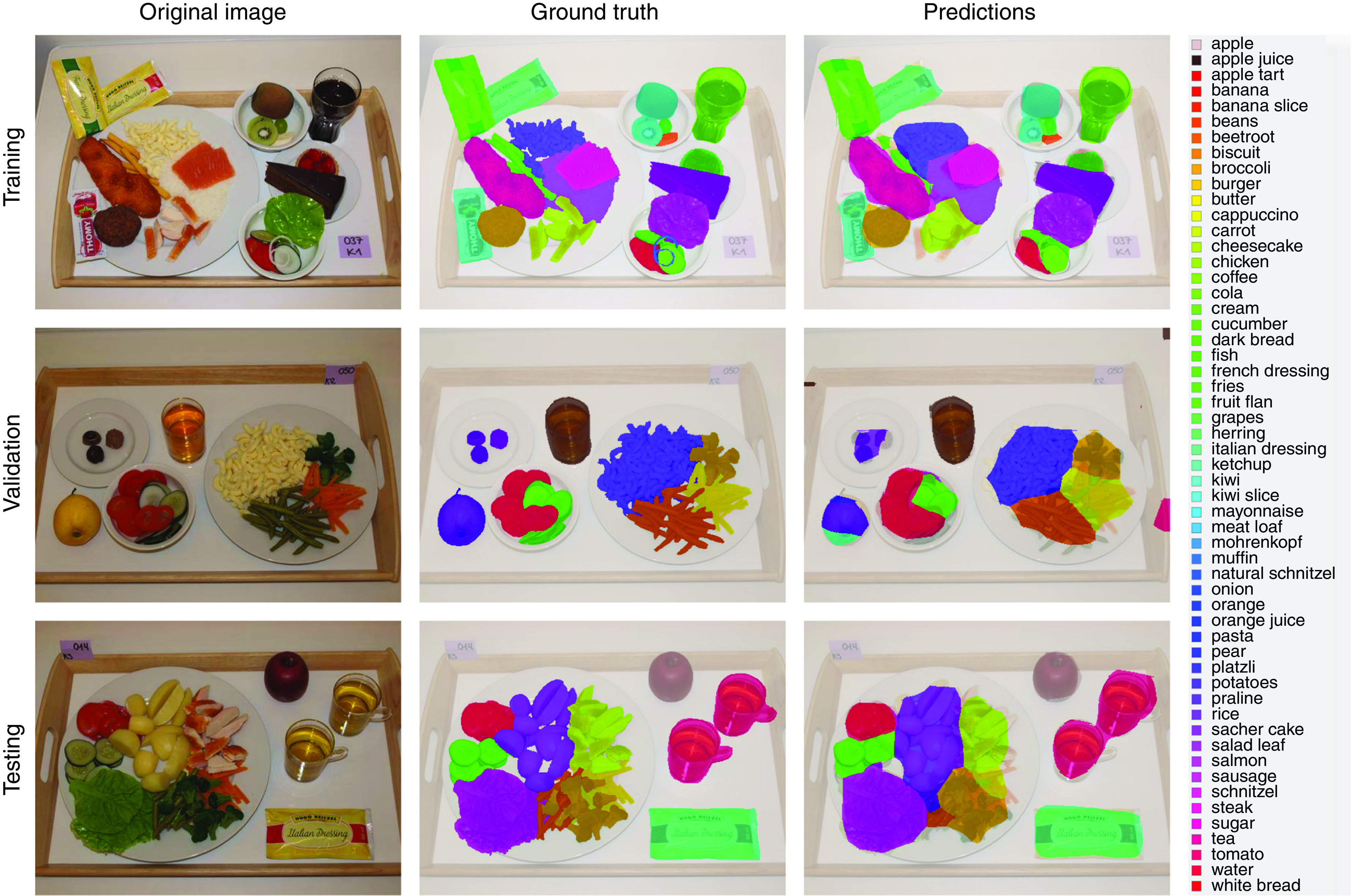
Example images from each of the three subsets (training, validation and testing) of the fake food buffet data set, along with the corresponding ground-truth label images. The third image column contains predictions from the FCN-8s deep learning model. Each colour found in the images represents a different food or drink item; these items and their corresponding colours are listed to the right of the images

The training of the FCN-8s deep learning model took approximately 37 h of computation on the previously mentioned graphics processing unit. Classifying a single image, however, takes significantly less time and computing power, which makes the use of deep learning models possible even in mobile applications. After the training was completed using the training and validation subsets, the model was run once on the testing subset. This generated label images for the fake-food images, which were then compared with the ground-truth label images using the measures mentioned above. Table 1 contains these results, whereas Fig. 2 contains three example images (one from each subset) with the corresponding ground-truth and model prediction labels.

**Table 1:** Results from the FCN-8s deep learning model

	Pixel accuracy (%)	Mean accuracy (%)	Mean IU (%)	Frequency-weighted IU (%)
Training	93·43	81·51	72·74	89·09
Validation	90·41	65·12	55·26	84·86
Testing	92·18	70·58	61·85	87·57
All	93·33	80·78	71·99	88·95

IU, Intersection over Union.

As expected, the performance of the FCN-8s model was better on the training subset than on the other two subsets. However, the difference is not substantial, which means the model learned features that generalize well. It is important to note that this performance was measured on all classes; this includes the background, which represents the majority of the pixels. Since the testing subset contains images new to the deep learning model, we consider the results on this subset to be the most representative of real-world performance. Out of these results, we chose pixel accuracy as the final quality measure, since this measure is analogous to the classification accuracy in the traditional convolutional neural networks that classify an entire image into one class. The difference is that instead of computing accuracy on an image level, it is computed on a pixel level. As can be seen from Table 1, the final accuracy for our FCN-8s deep learning model was therefore 92·18 %. Additionally, the ratios between the quality measures seem consistent with those of Long *et al.* ^(^ ^34^ ^)^.

Due to the higher accuracy, the predictions for the training subset offer more detail than those for the other two subsets and are very close to the ground truth, with the only exception being very small food items, such as onion rings, as can be seen in the training predictions image in Fig. 2. However, despite the lower amount of detail, the majority of the predictions for the other two subsets are still accurate. There are some misclassifications in the data set, such as parts of the pear and small parts of the background in the validation predictions image in Fig. 2, but these errors are rare. A more common occurrence that lowers accuracy is when the predictions do not cover the food and drink items exactly.

### Results from food matching and standardization

To support the process of automated dietary assessment, each fake-food item needs to be automatically matched to nutrient data from an FCDB.

The result for each fake-food item obtained using the deep learning model is one of the fifty-five foods (food classes) for which the model is trained and is used in the FFB method. In this task we used StandFood to standardize each food class that results from the deep learning model. For this reason, we used the English names of the fifty-five food classes. First, for each food class, the classification part of StandFood is used to obtain its food category (raw, derivative, simple or aggregated composite food). The food class is also used with the description part to obtain its list term (i.e. the FoodEx2 identifier). After these two parts, their results are combined to improve the classification of the food class, in case the model used in the classification part incorrectly classifies it.

Table 2 presents the results from the StandFood classification part of four randomly selected but correctly classified food classes, one per food category. The StandFood classification part has an accuracy of 75 %. This is further improved using the StandFood post-processing part, but before we used it, the result from the description part needed to be obtained.

**Table 2:** Correctly classified food classes using the StandFood classification part and description of the food classes using the StandFood description part

Correctly classified food classes using the StandFood classification part	
Food class (result from the deep learning model)	StandFood food category (according to FoodEx2)
Broccoli	Raw (r)
Sugar	Derivative (d)
Pasta	Aggregated composite (c)
White bread	Simple composite (s)
Description of food classes using the StandFood description part	
Food class (result from the deep learning model)	StandFood relevant FoodEx2 item and its descriptor
Apple	Apples (A01DJ)
Biscuit	Biscuits (A009V)
Sugar	White sugar (A032J)
	Brown sugar (A032M)
	Flavoured sugar (A032Q)
	Sugars and similar (A0BY6)
Pasta	Fresh pasta (A007F)
	Dried pasta (A007L)

Concerning the second part, Table 2 provides the results from the StandFood description part of four randomly selected food classes, one for each food category. As can be seen, for the first two food classes we have perfect matches, while for the next two we have multiple choices. The multiple choices happened because of the food class description. For the last two examples provided in Table 2, the food class description is too general, so the StandFood description part suggests the most relevant matches to users. For example, for the food class ‘pasta’, the most relevant matches provided by StandFood are ‘fresh pasta’ or ‘dried pasta’. To distinguish between them in the process of automated dietary assessment is a really important task because they have different nutritional profiles. It follows that the description of the food classes that is the result of the deep learning model is the key to how successful the automatic food matching will be. In the present study, we evaluated the proposed methodology using the food classes described in the FFB method. The StandFood description part has an accuracy of 86 %. In the 14 % that are not correctly described, this is caused by some culture-specific foods or food classes for which the StandFood description part could not find nouns in their description. This happened because the StandFood description part uses the extracted nouns from POS tagging for each food class, and to produce its relevant match the FoodEx2 data are searched for the names that consist of at least one of the extracted nouns. In cases when nouns are not found in a food class description, the description accuracy increases to 93 % by using the extension of the description part. Two randomly selected examples in the case of fake foods when this happened are for the food classes ‘French dressing’ and ‘herring’. After the POS tagging, ‘dressing’ and ‘herring’ were not recognized as nouns and the StandFood description part did not provide a result. However, this was solved using the Levenshtein distance between the food class and each description presented in the FoodEx2 data. In the examples of ‘French dressing’ and ‘herring’ this returned ‘salad dressing’ and ‘herrings’.

In addition to the FoodEx2 identifier, the StandFood description part returns the FoodEx2 food category of the most relevant match. This is further combined and used in the post-processing rules together with the food category obtained by the StandFood classification part to improve the classification accuracy. Table 3 presents the results of three randomly selected food classes after the post-processing part. After the post-processing part, the classification accuracy increases to 93 %.

**Table 3:** StandFood post-processing result of three randomly selected food classes

Food class (result from the deep learning model)	StandFood classification food category (according to FoodEx2)	StandFood post-processing food category (according to FoodEx2)
Muffin	Raw (r)	Aggregated composite (c)
Praline	Raw (r)	Simple composite (s)
Coffee	Derivative (d)	Simple composite (s)

In addition, if we want to link these food classes to the FCDB, we need to search the FCDB for their FoodEx2 identifiers. If the FCDB lacks the FoodEx2 identifiers, StandFood can be used to find these identifiers and to describe all the food items that exist in it.

## Discussion

In the current study we have developed an advanced methodology for automatic food image recognition and the standardization of food items that supports the process of automated dietary assessment. The methodology was evaluated using food images collected using the FFB method.

Since this is the first automatic solution for recognizing the images of fake foods, we consider our results as a baseline for any future studies. Directly comparing our pixel accuracy with the classification accuracy results of other food image recognition solutions^(^ ^16^ ^–^ ^27^ ^)^ is not appropriate because not only were those solutions tested on different data sets with a different number of food classes, but there is also a difference in the performance measures used and in the image variance; fake food generally exhibits less variance than real food, as real food can be prepared in multiple ways, which can affect its visual appearance. There have been some food recognition solutions that apply pixel-level segmentation in the past, but only one that uses deep learning^(^ ^22^ ^)^. However, even that one uses manually defined feature descriptors for the segmentation phase and deep learning only for the classification, so to the best of our knowledge the present study is the first that applies a single deep learning network for the joint segmentation and classification of food items. The study’s results provide a base for an automated dietary assessment solution.

As the food-matching approach also enables the semi-automated assignment of food descriptors (with respect to the selected food classification and indexing system, such as FoodEx2), the linkage of food items with any FCDB complying with the selected food classification and indexing system can be performed.

Automation of the recognition of fake foods and matching them with information from a nutrient database offers great potential for research. In particular, it would reduce the effort to collect and analyse the data; that is, foods selected by participants can be assessed from photographs instead of by manual handling. In practice, the simplest approach would be to implement the solution proposed herein in a smartphone app, which would allow researchers to automatically gain relevant information about the selected foods by taking a photograph using the smartphone’s camera, thus allowing them to instantaneously analyse the data. This type of automation would also reduce the biases introduced by human errors in the data and would facilitate data standardization, comparison and exchange between different laboratories using this research tool. Research questions, such as which food groups were selected more often, could be investigated automatically. The matching also allows us to study patterns in food choice (e.g. which foods are selected in combination, etc.). It can also facilitate secondary data analysis on fake-food studies, where photographs have been taken. Photographs from different experiments and laboratories could be combined for this.

Future work includes an extension of this methodology with a tool that automatically measures weight (e.g. food scape lab), or a technology that automatically estimates food volume, as this is currently the only missing part in the process of automated dietary assessment. Although the predictions from the deep learning model for the validation and testing images are not as detailed as for the training ones, they still describe the food and drink items with an accuracy that could also be sufficient for a food and drink volume estimation when paired with either a reference object or a fixed-distance camera.
